# Extremely Preterm Neonate with a Tracheobronchial Foreign Body: A Case Report

**DOI:** 10.7759/cureus.7659

**Published:** 2020-04-13

**Authors:** Sreekanth Viswanathan, Yahdira Rodriguez Prado, Caroline Chua, Darlene A Calhoun

**Affiliations:** 1 Pediatrics, Nemours Children's Hospital, Orlando, USA

**Keywords:** premature infants, tracheobronchial injury, intubation stylet, foreign body, intubation complication

## Abstract

We report the case of an approximately 27-week gestational-age preterm infant admitted on the day of life number four for evaluation of a foreign body noted on serial chest X-rays. CT of the chest revealed a foreign body present in the trachea, extending from just above the tracheal bifurcation deep into the posterior basilar segment of the right lower lobe. Endoscopic removal of the foreign body revealed a portion of the plastic sheath of the stylet used during intubation. We also provide a brief review of the relevant literature.

## Introduction

Stylets are commonly used to facilitate endotracheal intubation of neonates in the delivery room, operating room, and neonatal intensive care unit (NICU). While several types of stylets are available for use, rare complications related to plastic-coated stylets or those covered by a plastic sheath have been reported [1-9]. In the majority of the documented cases, the endotracheal tube (ETT) was obstructed by the sheared-off portion of the stylet sheath. This is the first reported case of a delayed tracheobronchial tear caused by a dislodged plastic stylet sheath that accidentally sheared off during intubation in an extremely preterm infant. The plastic sheath was recovered using optical forceps.

## Case presentation

**Figure 1:**
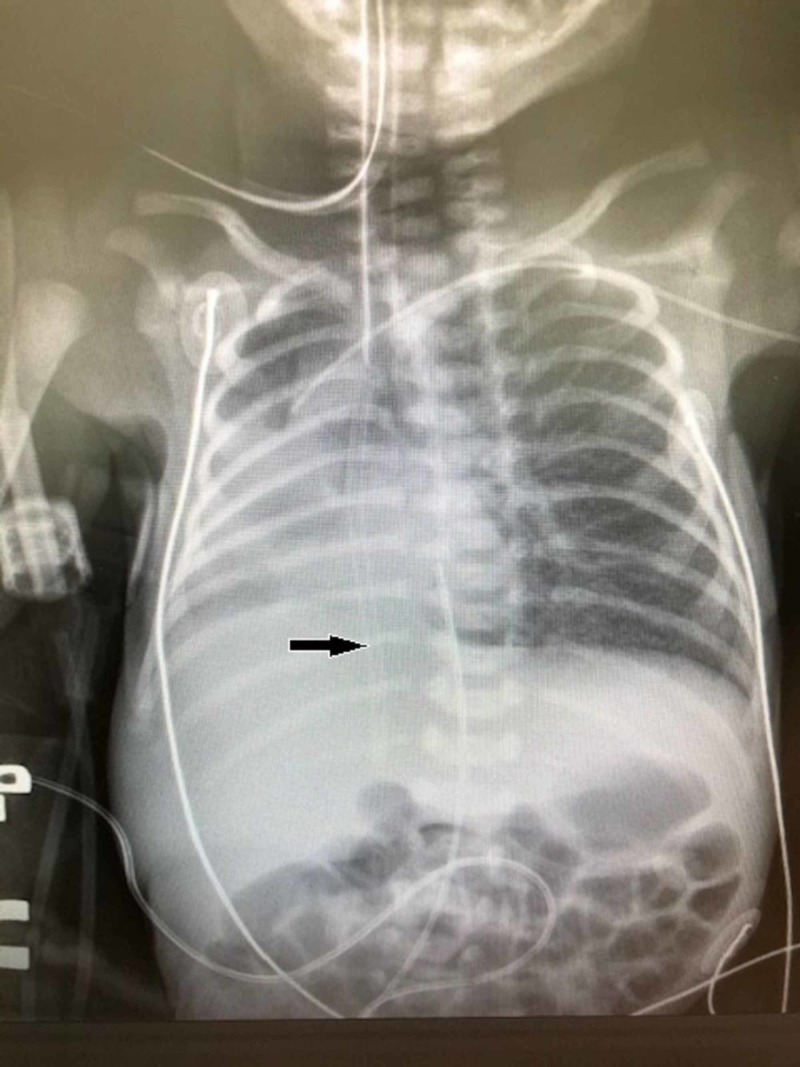
Chest X-ray of the patient obtained to verify PICC line placement The arrow notes the presence of a foreign body later found to be a portion of the plastic sheath covering the stylet used during intubation PICC: peripherally inserted central catheter

A 27-year-old female presented at 25 weeks’ gestation with premature rupture of the membranes. The infant was subsequently delivered at 27 weeks’ gestation by Cesarean section secondary to fetal decelerations and known breech position of the fetus. The male infant weighing 1,220 grams was intubated without difficulty with a 3-mm cuffless endotracheal tube with a 6 Fr Medline DYND43506 intubating stylet (Medline, Northfield, IL) in the operating room and was given a dose of Curosurf® (natural porcine lung surfactant; Chiesi Farmaceutici, Parma, Italy). He remained intubated and was given a second dose of surfactant on day of life two. On day of life three, he was extubated and placed on nasal continuous positive airway pressure (CPAP). On day of life four, a left antecubital peripherally inserted central catheter (PICC) was placed, and the subsequent X-ray to verify PICC placement noted right middle and right lower lobe atelectasis with shifting of the cardiac silhouette to the right (Figure 1). At the same time, a thin tube-shaped object was noted over the right lung field. A review of previous X-rays noted that the same object had been seen on the admission X-ray but had been considered an artifact. The infant was subsequently intubated and transferred to our center for further evaluation and management of the foreign body in the right lung.

**Figure 2:**
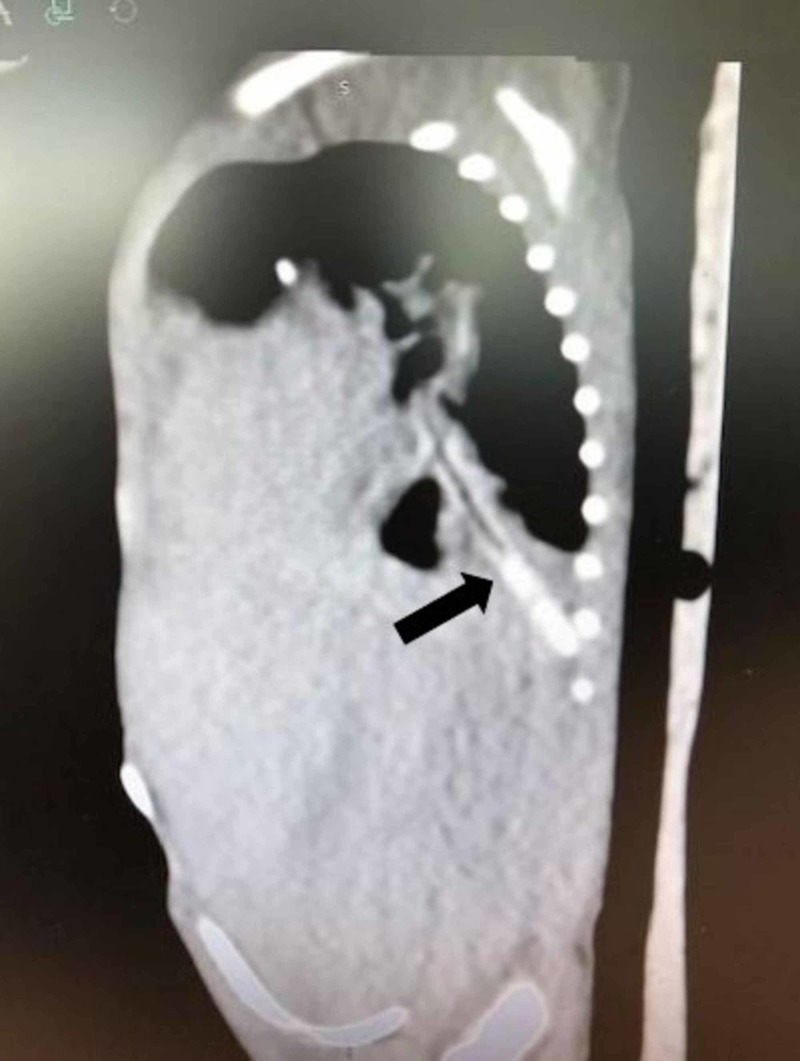
Sagittal view of CT of chest and abdomen The arrow notes the position of the stylet sheath on sagittal view of the patient's CT of the chest/abdomen. The sheath fragment extended into the most distal portion of the segmental bronchus to the posterior segment of the right lower lobe CT: computed tomography

Upon arrival, the neonate was hemodynamically stable and X-rays demonstrated a foreign body beyond the tip of the ETT projecting over the right thorax and right upper abdominal quadrant. CT of the chest and abdomen performed without contrast demonstrated that the foreign body was approximately 5 cm in length with its distal tip deep within the posterior segment of the right lower lobe and the proximal end at the most distal portion of the trachea just above the bifurcation (Figure 2).

**Figure 3:**
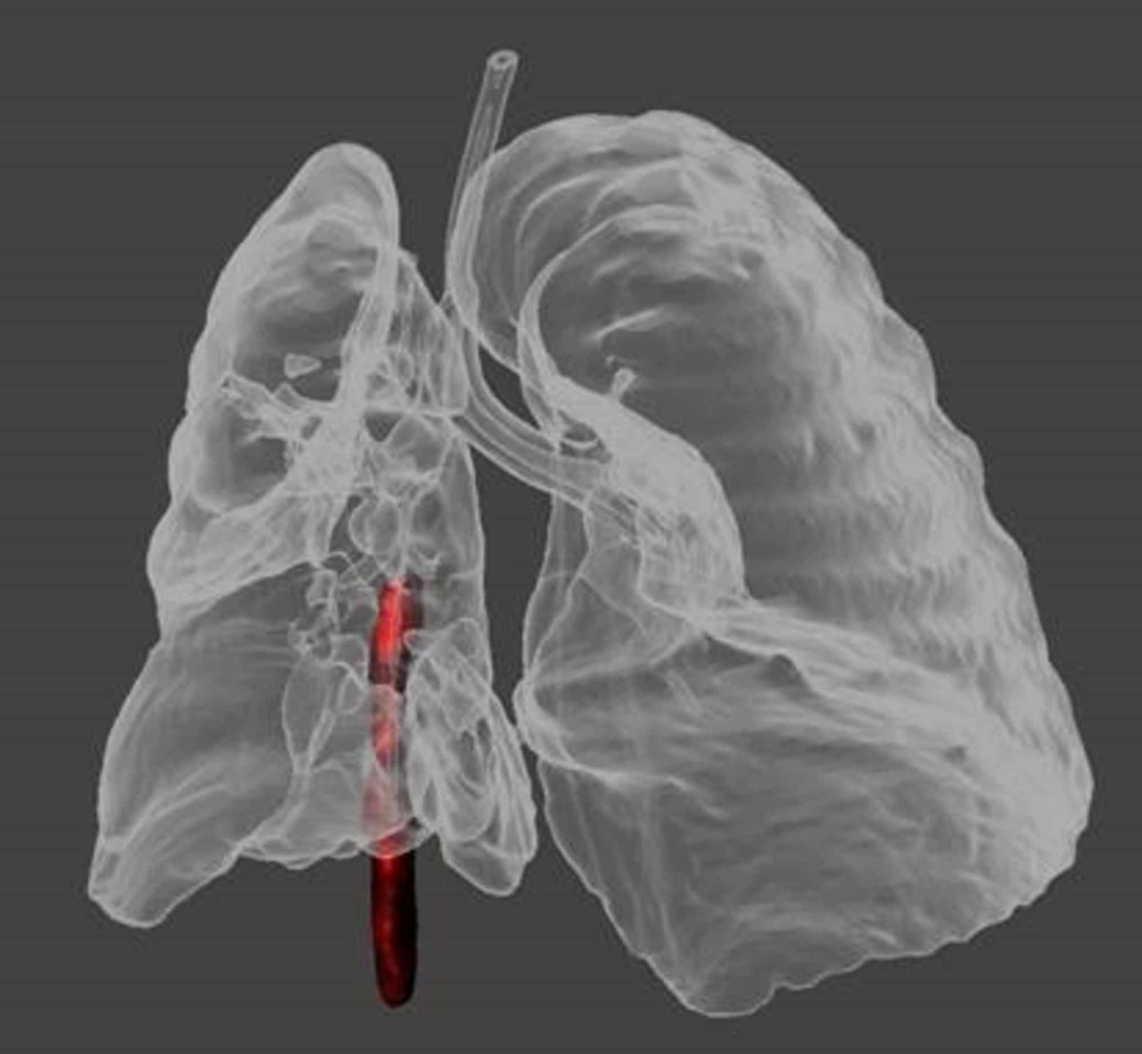
3D view of the chest CT - frontal projection Frontal projection of the 3D reconstruction of neonate's chest CT prior to removal of the foreign body (shown in red) CT: computed tomography

**Figure 4:**
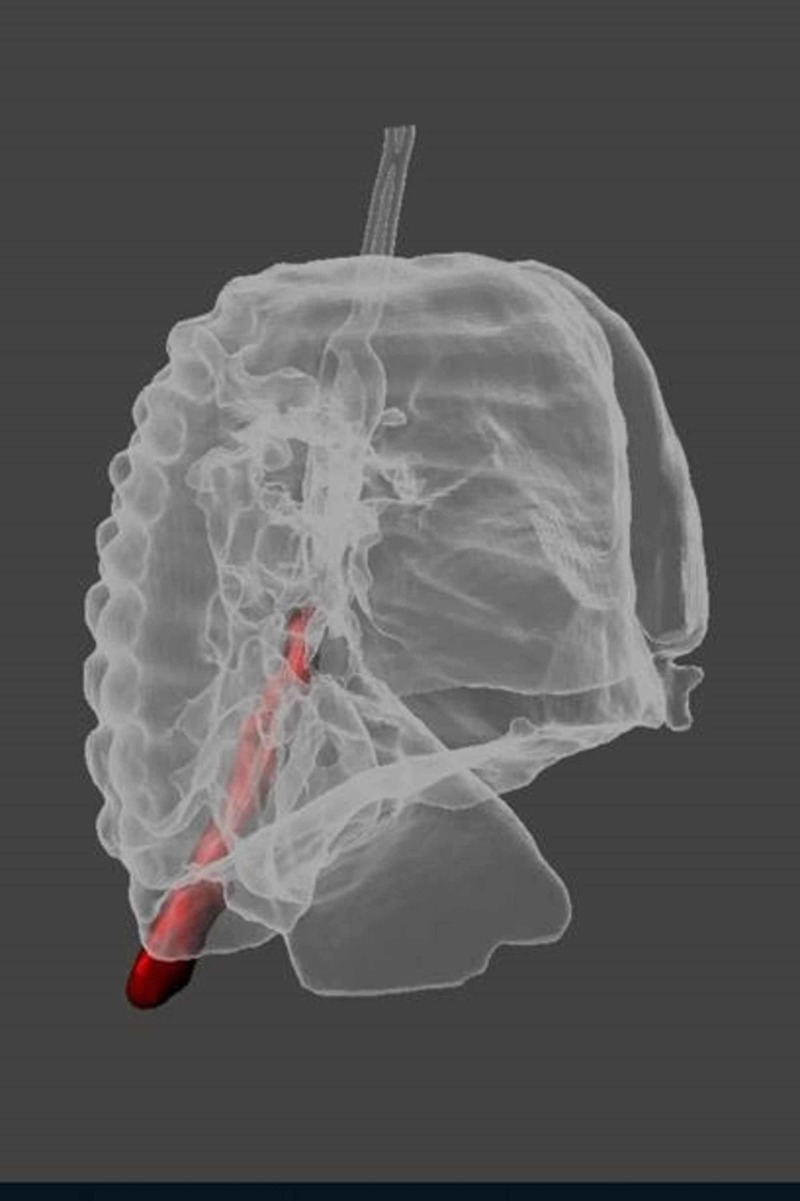
3D view of the chest CT - lateral projection Lateral projection of the 3D reconstruction of the chest CT demonstrating the position of the foreign body (red) prior to removal CT: computed tomography

The foreign body was noted to extend inferiorly, traversing through the right mainstem bronchus, bronchus intermedius, bronchus to the right lower lobe, and into the most distal portion of the segmental bronchus to the posterior segment of the right lower lobe. The tip was noted to be deep in the costophrenic sulcus (Figures 3, 4).

**Figure 5:**
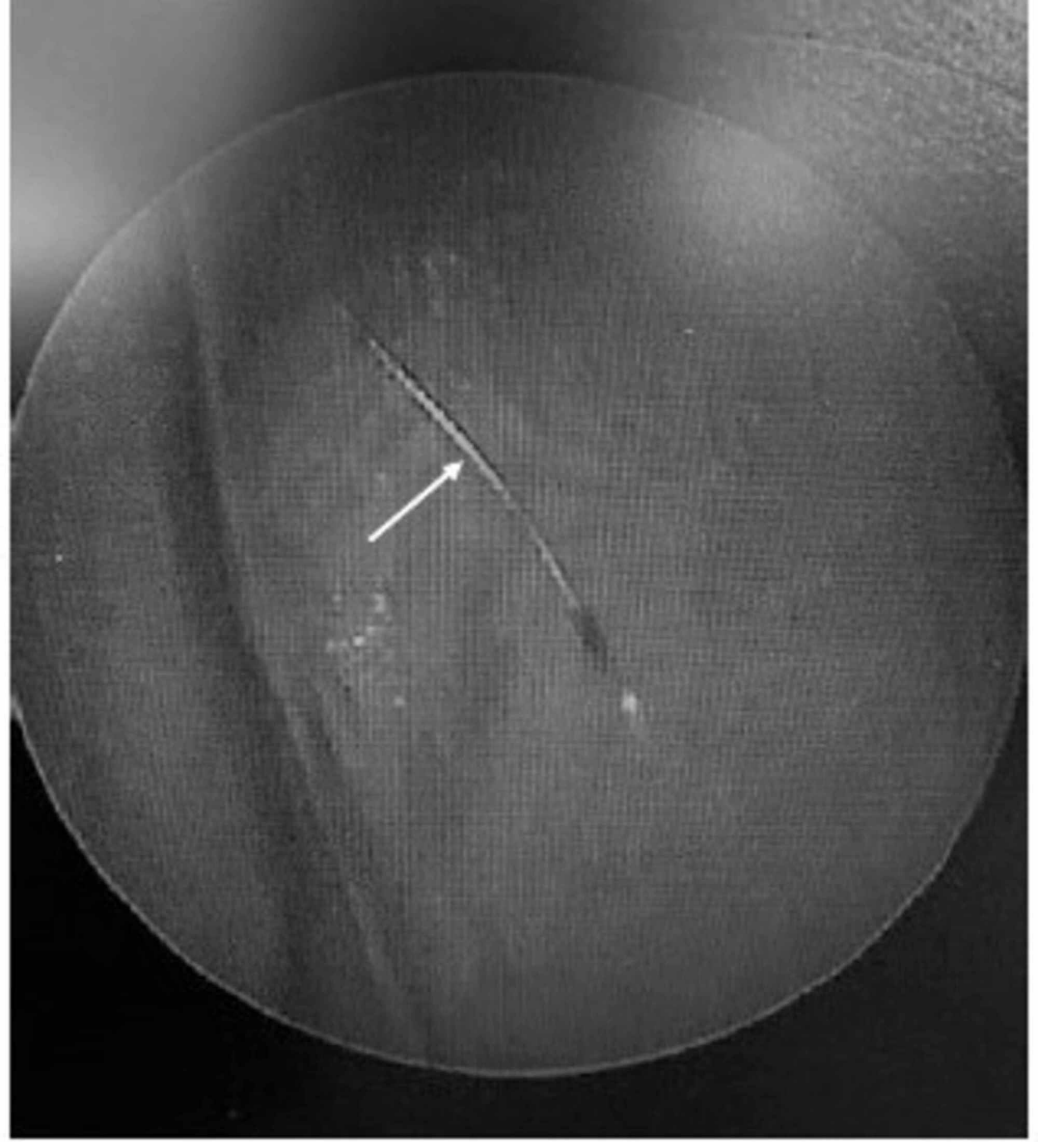
Plastic sheath of the intubation stylet The arrow notes the fragment of plastic sheath from the intubation stylet removed from the patient

Because the patient was hemodynamically stable, the pediatric surgical team in collaboration with otolaryngology developed a plan for the removal of the foreign body by direct visualization. The cardiothoracic surgery team was on stand-by in the event that an emergency thoracotomy was necessary if direct visualization was unsuccessful. The patient was scheduled for the removal of the foreign body the day after admission; however, within an hour of the CT scan, he developed active bleeding from the ETT. A STAT chest X-ray revealed that the foreign body had moved and was now adjacent to the distal tip of the ETT. The patient was taken emergently to the operating room on conventional ventilation; under general anesthesia, the otolaryngologist first visualized the airway using a 2.4-mm Hopkins telescope. The foreign body was visualized in the trachea. The telescope was then removed, followed by the removal of the existing ETT. Optical forceps were introduced and, with direct visualization, the object was removed revealing the sheared-off plastic stylet sheath used during the initial intubation of the neonate (Figure 5). The telescope was reintroduced for the inspection of the airway, and there was no residual foreign body, bleeding, or tears observed in the mucosa, the trachea, or main stem bronchi apart from areas of mild ecchymosis in the right main stem bronchus.

**Figure 6:**
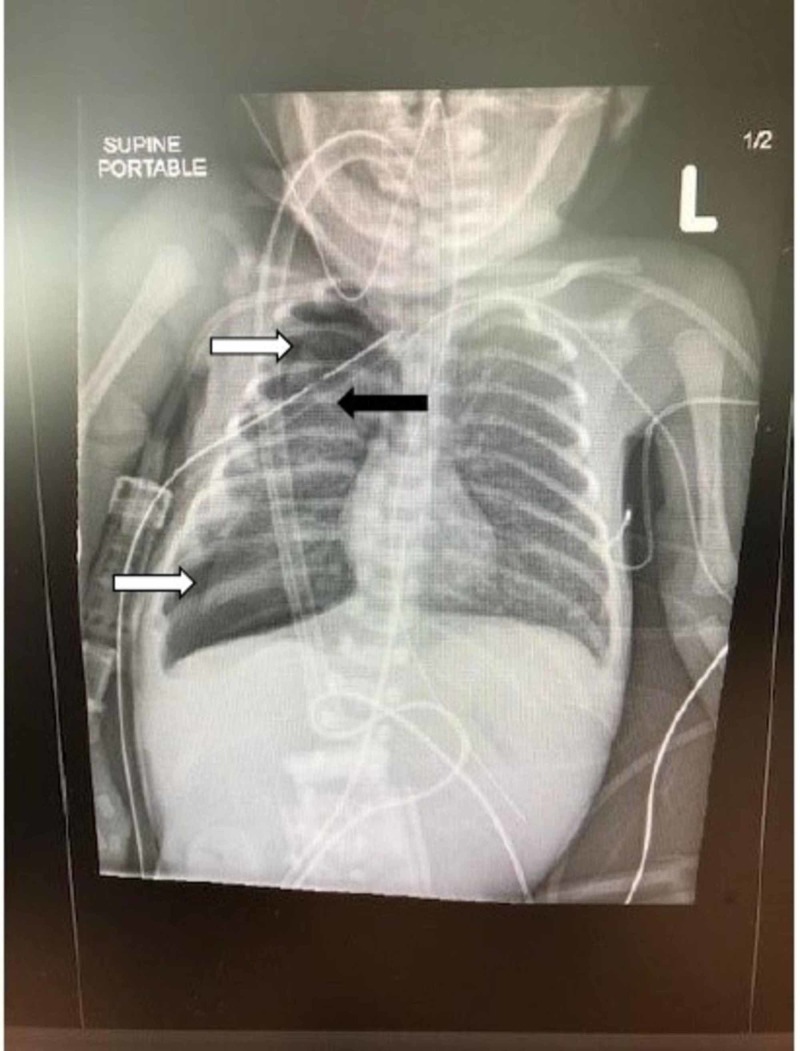
Chest X-ray obtained in the operating room Interval development of a right-sided pneumothorax (white arrows) occurred after the removal of the fragment of plastic sheath. Residual pneumothorax is demonstrated after placement of the right-sided chest tube (black arrow)

The patient was then intubated with a 3-mm cuffless ETT. However, the neonate rapidly developed signs of decompensation with hypoxia and decreased breath sounds on the right side, suggestive of a tension pneumothorax. Following chest X-ray confirmation of the pneumothorax, a right-sided chest tube was immediately placed with rapid improvement in the neonate’s condition (Figure 6).

The infant was returned to the NICU, and a continuous air leak consistent with a bronchopleural tear was managed with conventional mechanical ventilation. The patient required placement of a second chest tube within the next 48 hours due to the re-accumulation of the right-sided tension pneumothorax. At this time, the mode of ventilation was changed to high-frequency jet ventilation, which continued until the infant’s initial chest tube was removed on post-op day eight. The second chest tube was removed on post-op day 11, and the patient was then electively extubated on the same day to CPAP support. The respiratory support was further weaned to a high-flow nasal cannula on day of life 16 and finally weaned to unassisted room air on day of life 69. The infant was discharged home on day of life 86, at the postmenstrual age of 39.1 weeks.

## Discussion

Stylets are commonly used during neonatal intubation. These stylets are often plastic coated to prevent airway injury from the metal end and to facilitate removal after insertion of the ETT. A review of the relevant literature in English yielded nine published reports related to the shearing of the plastic sheath covering intubation stylets in neonates between 1985 and 2016 [1-9]. Most cases reported either a partial or complete obstruction of ETT by the sheared portion of plastic coating. In such cases presenting as acute airway obstruction, subsequent management led to the immediate removal of the foreign body when the ETT was removed. In two of the reported cases, the sheared stylet sheath did not obstruct the ETT but instead migrated into the trachea and the left main bronchus [2,8]. In both cases, fragments were able to be removed using direct visualization of the trachea [2,8]. The patient in this case represents an additional reporting of a rare complication of neonatal intubation involving further migration of the stylet sheath into the tracheobronchial tree resulting in a tracheobronchial tear and injury to the lung parenchyma.

In this case, the foreign body was considered an artifact when first noted on the patient's initial chest X-ray. At that time, the baby was noted to be doing well from a clinical perspective. Unfortunately, the foreign body was overlooked in the first three days but was subsequently noted on a routine X-ray for confirmation of PICC placement on day of life four.

Tracheal intubation is a common procedure for neonates in the NICU. In these patients, procedure-related complications are rare but can be severe, often resulting in respiratory compromise as illustrated in this case and other reports [1-9]. While it is not clear how the sheath sheared during this patient’s intubation procedure, others have reported that repeated bending of the sheath, reuse of a stylet, and excessive grasping of the tube while removing the stylet can result in a sheared fragment of the plastic sheath [1]. The sheared fragment may also be pushed in further during reintubation, potentially resulting in migration to a location where retrieval may be difficult and may lead to pulmonary parenchymal injury, as revealed in this case [2,8]. Emergency thoracotomy may be necessary in such situations; in this case, the potential need for such an intervention was anticipated. In addition, the persistent pneumothorax with a large air leak in this preterm infant posed the potential need for emergency thoracotomy if the patient's cardio-respiratory status worsened. The use of thoracotomy has been shown to be lifesaving in infants on ventilator support who continue to have a persistent pneumothorax unresponsive to chest tube insertion [10,11].

## Conclusions

The use of stylets during intubation is a common practice in neonatal patients and includes the use of plastic-coated intubation stylets. Caregivers should be aware that rare but potentially life-threatening complications can occur with the use of plastic-coated stylets. This case demonstrated an even rarer complication resulting from the migration of the sheared stylet sheath. The migration of the sheath fragment resulted in a potentially life-threatening event that initially went unnoticed for days following the intubation because the ETT was notobstructed by the sheared plastic sheath. Careful inspection of an intubation stylet with a plastic sheath should be performed before and after intubationprior to any application of positive pressure ventilation or administration of surfactant. In addition, the use of a new stylet with each intubation attempt and minimizing bending and rebending of the stylet should further reduce or prevent such iatrogenic complications.
